# Randomised controlled trial with parallel process evaluation and health economic analysis to evaluate a nutritional management intervention, OptiCALS, for patients with amyotrophic lateral sclerosis: study protocol

**DOI:** 10.1136/bmjopen-2024-096098

**Published:** 2025-05-27

**Authors:** Arron Peace, David Alexander White, Gemma Hackney, Mike Bradburn, Paul Norman, Sean White, Ammar Al-Chalabi, Wendy Baird, Daniel Beever, Janet Cade, Elizabeth Coates, Cindy Cooper, Naseeb Ezaydi, Vanessa Halliday, Chin Maguire, Pamela J Shaw, Haris Stavroulakis, Simon Waterhouse, Tracey Anne Young, Christopher J McDermott, on behalf of the HighCALS group, Elaine Scott

**Affiliations:** 1 Clinical Trials Research Unit, Sheffield Centre for Health and Related Research, The University of Sheffield, Sheffield, UK; 2 Department of Psychology, The University of Sheffield, Sheffield, UK; 3 Sheffield Teaching Hospitals NHS Foundation Trust, Sheffield, UK; 4 Department of Neuroscience, King’s College London, London, UK; 5 Sheffield Centre for Health and Related Research, The University of Sheffield, Sheffield, UK; 6 School of Food Science and Nutrition, University of Leeds, Leeds, UK; 7 Division of Neuroscience. School of Medicine and Population Health. Sheffield Institute for Translational Neuroscience, The University of Sheffield, Sheffield, UK

**Keywords:** Health, NEUROLOGY, Motor neurone disease, NUTRITION & DIETETICS

## Abstract

**Abstract:**

**Introduction:**

Amyotrophic lateral sclerosis (ALS) is a devastating illness that leads to muscle weakness and death usually within around 3 years of diagnosis. People with ALS (pwALS) often lose weight due to raised energy requirements and symptoms of the disease presenting significant challenges to taking adequate oral diet, with those who lose more weight being at a greater chance of earlier death. There is also some evidence to suggest that a higher calorie diet may benefit the disease course in pwALS, but further research is needed.

**Methods and analysis:**

Two armed, parallel group, superiority, open labelled, randomised controlled trial, with internal pilot, to assess the effectiveness of an early high calorie diet on functional outcomes in ALS, comprising two treatment arms: (1) standard care, (2) standard care with additional active management using the OptiCALS complex intervention to achieve a high calorie diet (initially randomised 1:1, then 1:2 following a protocol amendment). Using a food first approach, pwALS will be encouraged and supported to follow a diet that meets an individualised calorie target from food before prescribing oral nutritional supplements. 259 pwALS will be recruited from up to 20 ALS centres across the United Kingdom and Ireland and followed up for a period of 12 months. Primary outcome is functional change measured over 12 months, using the Revised Amyotrophic Lateral Sclerosis Functional Rating Scale. Secondary end points include measures of functional health, quality of life, calorie intake and weight, as well as time to gastrostomy and survival. A health economic analysis and process evaluation will also be undertaken. Participant recruitment is expected to complete in September 2025, and participant follow-up is expected to complete in September 2026. The results of this study are expected in March 2027.

**Ethics and dissemination:**

The trial was approved by Greater Manchester—North West Research Ethics Committee, reference 20/NW/0334 on 8 September 2020. We will publish the study findings in peer-reviewed academic journals and present at local, national and international conferences where possible.

**Trial registration number:**

ISRCTN30588041.

**STRENGTHS AND LIMITATIONS OF THIS STUDY**

- The study builds on 4 years of formative research exploring attitudes, as well as enablers and barriers, to nutritional support for people with amyotrophic lateral sclerosis (pwALS) developed with specialists, pwALS and their carers.
- The intervention is designed to be delivered in the UK National Health Service, by non-specialists enabling an easier roll-out into clinical services if found to be beneficial.
- The study allows data collection and intervention delivery remotely. This enables participants to access the intervention where it may not otherwise be feasible due to disability.
- Due to the nature of the intervention, it is not possible to blind participants and investigators to treatment allocation; however, blinded outcome assessors are used to facilitate remote data collection. The use of remote data collection may risk inaccuracies in some outcome measures (height, weight, spirometry).

## Introduction

 Amyotrophic lateral sclerosis (ALS), also known as motor neuron disease (MND), is a devastating neurodegenerative disorder. The annual incidence is 1.5–2.5 per 100 000 with usual survival of around 3 years.^1 2^ Affected individuals experience a progressive weakness of limb muscles, loss of speech, chewing and swallowing problems, eventually succumbing to the consequences of respiratory failure due to respiratory muscle weakness.^3^ There is no curative treatment for ALS.^4^ Riluzole, the only drug treatment to have an effect on survival, slows progression by approximately 3 months.^5^

Malnutrition and weight loss are well-recognised poor prognostic factors in ALS.^4 6 7^ Body Mass Index (BMI) is an independent predictor of survival in ALS, with higher BMI (ie, 30–35) having a protective effect and lower BMI associated with a worse prognosis.^8^ In patients with weight loss of 5% or more at diagnosis, there is a twofold increase in risk of death, compared with those who have lost less than 5% of their usual premorbid weight.^79^,^11^ This is compounded by hypermetabolism, with resting energy expenditure being significantly higher than in healthy individuals.^912^,^15^ Postdiagnostic changes in BMI have been shown to predict survival, with those with a slower decline in BMI or increase in weight showing better prognosis.^15 16^

Current guidance on nutritional management with regard to assessment of nutritional status, total daily energy expenditure (TDEE) calculations, appropriate dietary intake or oral nutritional supplementation is based on weak evidence. Surveys indicate a lack of knowledge in healthcare professions regarding the nutritional management of ALS.^17^ The poor evidence base may also help explain the poor nutritional outcomes observed in pwALS.^18^,^20^

Metabolic defects and an energy deficit are an early feature of ALS pathology and not simply a consequence of denervation and advanced disease.^1 21^ Encouragingly, correction of the energy deficit with a high-calorie diet extended life by 20% in an ALS mouse model.^22^ Moreover, in patients with ALS, a small pilot study indicated a potential survival advantage and tolerability of a high-calorie diet.^23^ Two recent randomised trials have investigated this further, but both studies have limitations. A single-centre randomised controlled trial (RCT) of 88 pwALS compared nutritional counselling (with or without a mHealth app) versus standard care.^24^ The study had low power and failed to show a significant difference in weight but did signal a potential benefit of increased calorie intake on functional decline. Ludolph *et al* recruited 201 pwALS to an RCT comparing a high-fat nutritional supplement with placebo.^25^ The study showed no overall benefit on survival but did detect a significant benefit in fast-progressing patients. It is not clear whether the intervention increased calorie intake as this was not reported. The study also experienced high drop-out. In addition, two recent small-scale systematic reviews with meta-analyses showed no significant difference in Revised Amyotrophic Lateral Sclerosis Functional Rating Scale (ALSFRS-R) in pwALS taking high-calorie supplementation compared with control groups. It was highlighted that studies included were insufficiently powered, and therefore the reviews concluded that a high-quality, large-scale RCT was required.^26 27^

There is a need for a high-quality, adequately powered RCT, comparing a complex nutritional intervention to standard care, in order to confirm the potential of a high-calorie diet to influence the disease course in ALS. The National Institute for Health and Care Research funded the HighCALS Programme Grant to meet this need. It comprises a comprehensive programme of research to develop and evaluate an intervention, OptiCALS, to increase calorie intake in pwALS. Earlier work packages developed and piloted the OptiCALS intervention.^1728^,^31^ The study described in this protocol will evaluate that intervention.

## Objectives

1. An RCT to determine the effect of the OptiCALS intervention on both clinical and participant-related outcomes.
2. A process evaluation to facilitate interpretation of the RCT results and offer insights about how best to deliver the intervention in the real world.
3. A health economic evaluation to assess the cost-effectiveness of the OptiCALS intervention relative to current standard treatments available in the UK National Health Service (NHS).

## Methods and analysis

### Trial design

Two-armed, parallel group, superiority, open-labelled, RCT, with internal pilot, to assess the effectiveness of an early high-calorie diet on functional outcomes in ALS.

### Adaptations in response to the COVID-19 pandemic

The OptiCALS study has undergone significant changes to make the study safe and deliverable during the COVID-19 pandemic. In the original study design, participants would have to attend hospital visits to enable all data collection, as well as the delivery of the study intervention. After a delay to study launch, the study was redesigned to enable participant consent, data collection and intervention to be delivered remotely. This allowed the delivery of the study in a COVID-19 safe manner. Where participants attend hospital during a data collection window, participant blood is also collected for analysis. As the intervention was designed to be deliverable online, there were no changes made to the intervention.

### Participant identification

Participants are recruited from up to 20 sites delivering care to people with ALS.

### Inclusion and exclusion criteria

#### Inclusion criteria for pwALS

1. Age 18 years or older.
2. Diagnosis of ALS by the Gold Coast criteria.
3. Within 2 and a half years of onset of muscle weakness.
4. Stabilised on riluzole for 1 month or not on riluzole.

#### Inclusion criteria for caregivers

1. Aged 18 years and over.
2. Primary informal caregiver of a person with ALS who has consented to participate in the trial (either living with the person with ALS or a close family member or friend).

#### Exclusion criteria for pwALS only

1. Comorbidity that would affect survival or metabolic state (eg, unstable thyroid disease or unstable diabetes mellitus).
2. Using a gastrostomy tube for feeding (those using a gastrostomy tube for fluid or medication are not excluded).
3. BMI ≥35 kg/m^2^.

#### Exclusion criteria for pwALS and caregivers

1. Lacking capacity to provide fully informed written consent, verbal consent (for those who cannot provide written consent), or consent via the use of a communication aid.
2. Previous participation in the HighCALS research programme.
3. Unable to understand written and spoken English.

### Participant approach and recruitment

A sample of pwALS will be identified by local hospital systems (notes, electronic systems) or through self-identification via study advertising (local posters/leaflets, social media, Motor Neuron Disease Association, patient organisation websites). Those pwALS who are potentially eligible will be approached with an introductory letter, participant information sheet and consent form either via post, during a face-to-face routine clinic visit, or via email/post during/following a routine remote clinic appointment. Those potential participants who sent the information via email or in the post will be followed up within a week by the researcher/healthcare professional (HCP) to discuss the study over the phone and answer any further questions. Example participant consent forms are attached as supplementary materials to this manuscript.

### Remote consent procedures

If a face-to-face visit is not possible, a remote consent appointment will be arranged to take place via video or phone call, where either: (1) consent will be collected electronically (by email); (2) full verbal consent will be audio recorded using an encrypted dictaphone, with the permission of the potential participant; (3) the paper consent form will be completed by the participant and returned by post; or (4) an independent witness will join the call and sign consent on behalf of the participant.

### Randomisation

Participants will be allocated to one of two groups: standard treatment; standard treatment plus OptiCALS intervention (originally in a 1:1 ratio, amended during the study to a 1:2 ratio). Randomisation is via a web-based randomisation system, employing non-deterministic minimisation by including a random element into the allocation algorithm. The minimisation factors will be:

- Study site
- ALSFRS-R trajectory since symptom onset (a measure of functional decline that can predict individuals with fast and slow disease progression)
- Riluzole use

The minimisation will be hosted by the Sheffield Clinical Trial Research Unit (CTRU) randomisation system, SCRAM—a remote secure server internet-based system. Access to the allocation list will only be granted to those responsible for cleaning the data and preparing unblinded data summaries during the recruitment and follow-up. For all other members of the trial team, the allocations will be concealed until recruitment and data collection are complete.

A CTRU statistician who is not otherwise associated with the trial will generate the allocation sequence. Named persons on the delegation log, who are trained in research procedures, will allocate participants by logging into SCRAM and entering basic demographic information.

### Blinding

The outcome assessors will be blinded to treatment allocation during the trial recruitment and follow-up. Site staff who are delivering the intervention will not be responsible for collecting study outcome measures. Any instances of unblinding of the outcome assessor will be documented within the case report form and be reported as a secondary outcome. Study statisticians, health economists and those collecting outcome data will remain blinded while the study is ongoing.

### Study Intervention

**Figure 1:**
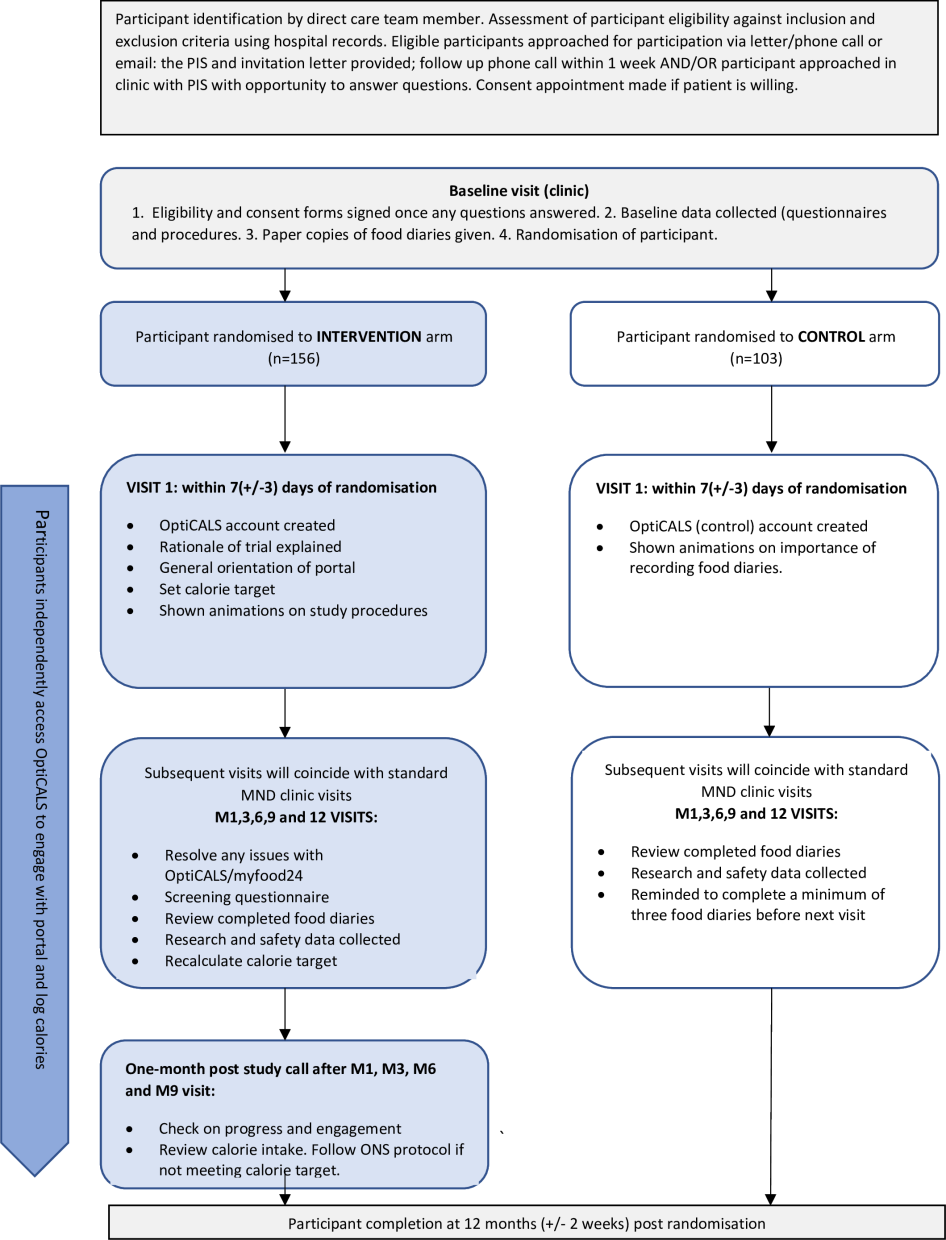
Patient flow throughout the study. MND, motor neuron disease; ONS, oral nutritional supplements; PIS, participant information sheet.

The OptiCALS portal will allow estimation of the participant’s TDEE using the ALS specific Kasarskis equation.^32^ At the first intervention study visit, an initial calorie target will be set based on the participant’s current calorie intake and their TDEE estimate (whichever is higher). At subsequent intervention study visits, the calorie target will be adjusted taking into account the participant’s current BMI, weight direction and TDEE estimate. Patients who are losing weight will be set a calorie target that is 400 calories above their current calorie intake, unless their current calorie intake is more than 400 calories below the TDEE estimate, in which case the TDEE estimate will be their calorie target. The study process is outlined in figure 1.

Participants will work with their HCP to develop a dietary strategy using a food fortification first approach (with or without oral nutritional supplements (ONS)) to meet their calorie target and in doing so, maintain or increase their weight. Specific areas of the portal provide information and resources for pwALS, carers and HCPs to help overcome barriers to eating a high calorie diet. These are summarised in table 1. The need for ONS and/or enteral feeding will be reviewed systematically throughout the study and discussed when participants are unable to meet their calorie target through a food first approach.

**Table 1:** Intervention modules

Module	Description
My food diary	Record dietary intake (a minimum of 3 days food diaries will be collected prior to each study visit to provide an estimate of current calorie intake). Estimate daily calorie intake.
How am I doing?	Presents participants with their individualised calorie target. Presents charts/graphs indicating current calorie intake vs calorie target. Presents charts/graphs of participants’ weight.
All resources	A range of resources and advice to help participants to meet their calorie targets, including animations, videos, text, PDFs and links to resources external to OptiCALS.
My resources	An area to save specific pages from within OptiCALS in one area. Individual pages on OptiCALS can be saved by the healthcare professional, participant and/or carer to the My resources page. Allow participants to write individual action plans about how they will meet their calorie target. Allow participants to view a list of saved ‘quick wins’, that is, foods and their calorie values that can be introduced to help them meet their calorie target.
Why boost my calories and weight?	Provides participants and carers with the rationale for why it is important to boost calories and increase weight in ALS.

ALS, amyotrophic lateral sclerosis.

The OptiCALS portal has five modules, with further subsections.

#### Intervention providers, support and training

RCT sites will allocate, or employ, members of staff from the ALS team to deliver the research and intervention procedures locally. Members of the central project team will support HCPs in the delivery of the intervention:

- All HCPs delivering the intervention will attend a training session run by the central project team before they are able to deliver the intervention to trial participants.
- Ongoing ad hoc support in answering questions, for example, how to tailor the intervention to participants, via an online discussion group embedded into the HCP area of the OptiCALS portal.

Training will include a face-to-face or virtual (if necessary) session where RCT HCPs will be orientated to the portal from logging on through to discussing calorie needs and tailoring the intervention. A user guide for HCPs will form part of the materials they use to facilitate intervention sessions with trial participants. HCPs will complete session logs to ensure delivery of the intervention as intended and these will both facilitate tailoring the intervention to individual participant needs as well as ensuring ongoing fidelity monitoring. Participants without access to the equipment necessary to engage with the OptiCALS intervention (ie, an internet-enabled device) will be provided with these, including access to a video call enabled device where clinic visits are not possible.

#### Intervention procedures: overview of visits

The intervention will be delivered by the HCP primarily during face-to-face sessions if possible, or via telephone/video calls if necessary over the course of the participant’s involvement, from the first intervention visit to 12 months postrandomisation.

Prior to starting the intervention, both intervention and control participants will meet with the food diary reviewer to be trained how to log food diary entries on to the myfood24 system area of the portal using three food diaries that they will have been asked to complete before the intervention visit. They will also be familiarised with the portal, ahead of their first visit.

#### Intervention visit 1: week 1

The first intervention visit will take place approximately 1–2 weeks after recruitment into the trial and will be arranged at a convenient time and place (via telephone/video call if necessary) for the participant and carer, with sufficient time to carry out the tasks (approximately 2 hours). The HCP will log in to the participant’s account. At this visit, the key tasks will be to generally orientate the participants and/or carer to the modules within the portal but, more importantly, to explain the rationale for the trial, that is, the importance of increasing calorie intake to maintain or increase weight.

The HCP will direct the participant to the ‘How I am doing: calories?’ page of the website to view their current calorie intake. The HCP will then complete the ALSFRS-6 questions with the participant to calculate the participant’s TDEE using the ALS-specific Kasarskis equation, which is embedded into the HCP part of the OptiCALS portal. This (or the participant’s current calorie intake, if higher) will be used as the initial calorie target. Participants will be directed to the ‘Calorie Boosting Quick Wins’ page and encouraged to identify foods that could be easily added to their daily diet to help them meet their calorie target. The participant will be asked to complete, and enter, another three food diary entries before their next intervention visit.

#### Intervention visit 2–5: months 1, 3, 6 and 9

At subsequent intervention visits or video/phone calls, the researcher/HCP will review the three food diaries completed by the participant and ensure they provide an accurate record of what they ate and drank. These visits will last approximately 1 hour. The HCP will complete the ALSFRS-6 questions again and enter the participant’s current weight. This information, along with the participant’s current calorie intake, will be used to adjust the participant’s calorie target.

Any issues relating to the accessibility and use of myfood24 or the OptiCALS website will be discussed by the HCP delivering the intervention, and support will be given to resolve these. The HCP will direct the participant to the ‘How I am doing: calories?’ page to identify if there is a calorie deficit and then view the ‘Calorie Boosting Quick Wins’ or discuss the use of ONS to bridge any calorie gap, as appropriate to each participant. The HCP will also complete a screening questionnaire which will suggest specific pages that can be saved to the participant’s ‘My Resources’ section of the OptiCALS website to help them boost the number of calories in their diet and/or manage common barriers to eating a high calorie diet. Finally, participants will be informed that they will receive a follow-up call in a month and will be asked to complete, and enter, three additional food diary entries in the week before the follow-up call. Onward referral for dietetic advice, gastrostomy and speech and language therapy will be as per standard clinical practice. The principal investigator at each site will communicate with the dietitian the rationale for the trial and request that they attempt to meet the calorie target set in OptiCALS when devising dietetic treatment plans. If participants are required to start ONS as a result of their inability to meet their calorie target, then they will initially be provided with a sample pack to try before an order is submitted. This will be followed up by a brief phone call from the HCP to check that they are happy with the ONS products provided.

#### Follow-up phone calls after intervention visits 2–5: months 2, 4, 7 and 10

All participants will receive a follow-up phone call from their HCP 1 month after each intervention visit to review their current calorie intake and any changes that have, or haven’t, been made to meet the new calorie target. Participants will be asked to complete, and enter, three additional food diary entries in the week before their next intervention visit.

#### Final intervention visit: month 12

At the final intervention visit, the researcher/HCP will review their final three food diaries. Participants will continue to be able to access the OptiCALS portal until the study ends. However, following the end of the 12-month trial period, no further calorie targets will be set by the supporting HCPs. Also, the HCPs will only continue to discuss the use of OptiCALS in clinic visits if they feel they have the resource to provide this support. There will be no expectation for the HCPs to continue to provide this support, and they will no longer receive excess treatment costs for this time.

The participant’s general practitioner (GP) and neurologist will be notified with an end of trial letter. If the participant is taking ONS at the end of the trial period, the PI will write to the participant’s GP and neurologist (and dietitian if involved), advising them that the participant has finished the trial and informing them of the specific ONS they have been taking, including the dose and calorie value, so that the same supplement or a similar product can be continued as part of standard care.

### Usual care

Usual care participants will receive the current standard nutritional management at each study site. Prior to their first visit, they will receive training on the myfood24 system. They will complete the myfood24 diary data through the ‘My food diary’ module, the only section of the OptiCALS portal available to the control arm participants. OptiCALS intervention modules will not be available to control arm participants, carers and other HCPs at the sites and, as such, will not be used to influence dietary management.

## Study outcomes

Participants will be followed up at week 1, month 1, month 3 and then at 3-month intervals until month 12 or until death if this is earlier. Survival status of each participant will be reassessed at the end of the study (between 12 and 35 months postrandomisation depending on when the patient was recruited). All outcome measures will be collected by a blinded assessor, except blood samples, calorie intake, adverse events (AEs) and acceptability of the intervention. Where clinic visits are not possible (eg, if shielding due to COVID-19), data will be collected at the participants’ home, either in person (if feasible), via video call, or via phone call if a video call is not possible. Where physical measures are to be collected via video or phone call, participants will be sent any equipment needed to complete these in advance of the appointment, as well as being directed to information about taking the measures in a separate section of the OptiCALS portal, including step-by-step videos. Training will also be provided by the blinded assessor prior to any measurements being taken. If neither a face-to-face nor remote follow-up appointment can be arranged, the questionnaire outcomes ALSFRS-R, WHOQOL-BREF, EQ-5D-3L, Healthcare Resource Use and Acceptability Questionnaire (intervention group only) can be self-completed by post or electronically via email.

### Primary outcome

Functional change over 1 year as measured longitudinally by the ALS Functional Rating Scale (ALSFRS-R),^33^ a validated rating instrument for monitoring the progression of disability in patients with ALS that correlates with survival and quality of life.

### Secondary outcomes

#### Combined Assessment of Function and Survival (CAFS)

This analysis ranks clinical outcomes on the basis of survival and change in ALSFRS-R scores.^34^ Each patient’s outcome is compared with every other patient’s outcome, assigned a score, and the summed scores are ranked. The mean rank score for each treatment group can then be calculated.

#### Quality of life for patients: WHOQOL-BREF

A 26-item quality of life measure developed by the World Health Organisation and validated for use in pwALS.^35^

#### Health status: EQ-5D-3L

A standardised measure of health status, covering five areas with three scoring options for each, developed by the EuroQol Group in order to provide a simple, generic measure of health for clinical and economic appraisal.^36^ Preference-based utility weights will be applied to EQ-5D-3L responses using UK population-based values from the UK value set.^37^ Utility values range from 1-perfect health to 0 death, with negative scores indicating states worse than death. This will be completed by both patients and carers.

#### Respiratory function: slow vital capacity (SVC)

A measure of respiratory muscle strength shown to correlate with survival in ALS, taken by spirometry. Forced expiratory volume in 6 seconds (FEV6) will also be taken via spirometry. Participants will be supplied with handheld spirometers. Participants who are able to use a smartphone-reliant spirometer will take SVC and FEV6 measurements. Participants who cannot use a smartphone-reliant spirometer will take FEV6 measurements only. Both measurements are considered acceptable alternatives based on existing literature.^38^,^42^

#### Anthropometric assessments

Triceps skin fold, mid arm circumference and calf circumference measurements will be taken using standardised methods.^43^ Given the asymmetry in ALS, the mean values taken from both upper and lower extremities will be used. Fat mass and fat free mass will then be estimated. If outcomes are measured remotely, triceps skin fold will not be taken and calf circumference will be used as the primary method of fat mass and fat-free mass estimation.

#### Overall survival

Defined as time from randomisation to date of death. Surviving patients will be censored at the date last known to be alive.

#### Total calorie intake

Self-completed by participants via myfood24.org (accessed via the OptiCALS portal). Participants will be asked to complete, and enter, three food diary entries in the week before each study visit. Access to an internet-enabled device will be provided for participants if needed.

#### Change in weight

% weight change throughout the study period.

#### Change in BMI

The BMI calculation divides an adult’s weight in kilograms by their height in metres squared. An existing record of measured height will be used if available in the patient notes. If no measured height is available, this will be taken remotely.

#### Time to gastrostomy

Defined as time from randomisation to date of gastrostomy procedure. If no procedure is recorded patients will be censored at the date last known to be without gastrostomy.

#### Acceptability of the intervention (intervention group only)

Self-reported using a bespoke 11-item questionnaire structured around the Theoretical Framework of Acceptability.^44^

#### Healthcare resource use

A questionnaire to measure use of healthcare services, used in the calculation of the cost-effectiveness of the intervention.

### Adverse events

#### Blood tests

Fasting lipids, albumin, lactate, renal function, thyroid function, neurofilament (Nf) serum, creatinine and C-reactive protein. If face-to-face visits are not possible, these measures will not be taken. The funding for analysis of Nf samples is via separate charitable funding, and as such, samples may be stored and analysed centrally beyond the completion of the trial.

### Safety

AEs may be identified at any point during the study via routine follow-up, contact from the participant and/or carer, on-site or remote monitoring or other sources (medical notes, etc). Participants and/or carers can inform site HCPs of adverse events at any point of the follow-up period. Related serious adverse events (SAEs) will be reported to the Sponsor, Research Ethics Committee and the Project Steering Committee providing safety oversight for the study.

There are no AEs excluded from collection although expected events related to ALS disease progression will not be reported as an SAE, unless the event is more severe than would normally be expected for a participant or if the investigator considers there to be a causal relationship between the trial intervention and the event.

### Participant retention

Participant retention is promoted through regular communication from the research team which clearly explains the importance of completing outcome data regardless of study arm. This message is reinforced at enrolment and all follow-up points. A flexible approach to study visits and delivery of intervention also aims to maximise participant retention, including a series of withdrawal options aiming to minimise participant burden, while maximising retention and data collection. Where participants are finding participation in the study challenging, but otherwise want to continue in the trial, options for reduced data collection throughout the remainder of the study will be discussed.

### Intervention completion and withdrawal

Study completion is defined as 12 months after the date of randomisation. There are three withdrawal options available to participants:

1. Withdrawal from the OptiCALS intervention only, that is, no further intervention delivery visits but the participant remains in the trial. The participant and/or clinician can make the decision to discontinue the intervention for any reason.
2. Withdrawal from the OptiCALS trial, but with consent for the collection of essential routine data from hospital notes, that is, survival status/cause of death and ALSFRS scores, where available. No further intervention or research visits will be conducted with the participant.
3. Withdrawal from the OptiCALS trial entirely, with no consent given to collect essential routine data. Unless specified by the participant, all data collected up to this point will be used in the analysis and no further intervention or research visits will be conducted.

Where participants have withdrawn, but consent to routine clinical data being collected, ALSFRS-R will be collected from their medical records along with survival status.

### Uptake of intervention

The intervention comprises several components (see table 1), each of which can be measured by the number of times accessed and the time span over which patients continue to engage with it. Each component will be summarised individually, and visual displays will depict how each individual engages with each component across time. For purposes of the analysis, these will be used to provide an overall assessment (blind to outcome) of whether each participant had engaged sufficiently to be considered ‘compliant’ with the intervention.

### Sample size

The sample size was derived by simulation with parameters estimated from the PRO-ACT ALS clinical trials data repository.^45^ The ALSFRS-R at entry was assumed to have a mean (SD) of 37 (6) and a mean monthly decline of 1 unit over the 12-month duration of follow-up, with successive values following with an autoregressive (AR1) correlation structure with rho=0.9. A difference of +0.25 units per month (ie, a monthly decline of 0.75 in the intervention arm vs 1.00 in the control arm) was taken as a clinically meaningful effect. The probability of death was assumed to depend on the last known ALSFRS-R with the probability of death in each 3-month period assumed to be zero up to month 3 and then as 91*exp[−0.115*(ALSFRS-R at previous visit)−3.95] thereafter, equating to an 18% death rate for the control group and 15% in the intervention at 12 months. These parameters were based on fitting a Cox regression model for survival in which ALSFRS-R score was a time-dependent covariate. Participants who die are included in the analysis and allocated a functional score of zero. Withdrawal was assumed to occur at a rate of 5% per quarter in both groups, again starting after the month 3 visit. With a two-sided alpha of 5%, a power of 85% and with the first 100 participants allocated in a 1:1 ratio and the remaining participants allocated 1:2 (standard care vs OptiCALS intervention), this requires a sample size of 103 in the standard care arm and 156 in the OptiCALS intervention arm, n=259 participants in total.

## Data analysis

### Primary outcome

The primary outcome ALSFRS-R will be analysed longitudinally using a multilevel linear mixed effect model estimated by restricted maximum likelihood. The fixed effect covariates will be time, treatment and their interaction together with variables used in the minimisation process. The recruitment centre will be incorporated as a random intercept in which participants are nested within their centre. If this model fails to converge, the centre effect will be removed and the simpler model will be fitted. The area between the two ALSFRS-R curves will be derived using a linear combination of the coefficients for treatment and the interaction of treatment with time and will be presented alongside its 95% CI and p value. The difference in ALSFRS-R will also be quantified separately at each timepoint.

The primary analysis will be intention-to-treat and will include all patients for whom data are collected or who have died; an ALSFRS-R functionality score will be assigned for all timepoints scheduled after the date of death. Sensitivity analyses will be performed to assess the robustness of the findings to distributional assumptions, baseline characteristics and imbalance, missing data and non-adherence.^46^

### Secondary outcome

Secondary outcomes will be analysed and reported in the same manner as the primary outcome, with the exception of time to event outcomes which will be analysed using Cox regression, and the combined analysis of function and survival which is a rank-based (non-parametric) test described in Berry *et al*.^34^ Analyses will be performed on an intention-to-treat basis, and all statistical tests will be two-tailed at 5% significance level. Further details will be provided in a full statistical analysis plan.

### Process evaluation

Alongside the RCT, a mixed-methods process evaluation will be undertaken. This includes qualitative interviews with pwALS, their carers and HCPs to explore context, implementation and mechanisms of impact. Where possible, we will approach patients and carers for separate interviews; however, if preferred, they will be given the choice to interview together, for example, to support with speech difficulties. Analysis of a sample of audio-recorded consultations and HCP intervention visit logs will be used to assess fidelity of intervention delivery. In addition to this, quantitative data will be collected using surveys administered to the intervention arm to investigate the acceptability of the intervention and data analytics on intervention usage.

All interviews will be transcribed verbatim for analysis. Observation notes will be written up after each visit, and reflexive notes will be written up after each qualitative interview to help with data interpretation. The qualitative data will be analysed thematically,^47^ within the broader framework for process evaluations recommended by the Medical Research Council.^48^ To understand the wider implementation issues, the analysis will draw on Normalisation Process Theory^49^ and the Theoretical Framework of Acceptability.^44^ To consider influences on the behaviour of individual pwALS, the COM-B model will be used as an overarching theoretical framework. NVivo software (QSR International) will be used to help structure the analysis.

### Health economics

A cost-utility analysis will be undertaken using the resource use, EQ-5D-3L and mortality data from the trial. The analysis will take an NHS and a broader societal perspective, with an additional analysis that incorporates carer quality-adjusted life years within the incremental cost-effectiveness ratio. This will be supplemented with decision analytic modelling to estimate lifetime cost-effectiveness for the patient cohort recruited to the study.

### Patient and public Involvement

#### Patient and public engagement

Patients and the public have been involved in all aspects of the study. The OptiCALS clinical trial builds on the back of 5 years of work (HighCALS programme) designed to understand the current national service models, the beliefs of stakeholders and the development of behaviour change strategies to support and improve nutritional interventions. The OptiCALS trial has been developed in collaboration with a variety of stakeholders including clinicians, nutritionists, psychologists as well as people living with MND, and their carers. This includes early consultation on study design, outcome measures and recruitment strategies. The OptiCALS Program Steering Committee consists of clinicians, methodologists and PPI members and meets regularly to offer continued input into the conduct, analysis, publication and future work of the study.

## Ethics and dissemination

### Governance

Sheffield Clinical Trials Research Unit on behalf of the sponsor (Sheffield Teaching Hospitals NHS Foundation Trust) coordinates the trial. The chief investigator, project coapplicants, members of the data management team, sponsor, trial manager and other representatives from the Project Management Group, who oversee the operation of the trial and through which amendments will be communicated. The Project Steering Committee (PSC), comprised of clinicians, statisticians, trialists and PPI representatives, provides independent oversight. The independent PSC also undertakes the activities of the Data Monitoring Ethics Committee, providing data monitoring and oversight.

###  Ethical approval

The trial was approved by Greater Manchester—North West Research Ethics Committee, reference 20/NW/0334 on 8 September 2020. The committee will be notified of amendments to the trial as appropriate.

### ISRCTN registration

The program was registered on the ISRCTN registry on 7 December 20 reference: ISRCTN30588041. 20, reference: ISRCTN30588041.

### Dissemination

We will publish the study findings in peer-reviewed academic journals and present at local, national and international conferences where possible. We will publish a short summary of the results on the OptiCALS trial website, accessed by trial participants as well as relevant interest groups.

## Supplementary material

10.1136/bmjopen-2024-096098 online supplemental file 1

10.1136/bmjopen-2024-096098 online supplemental file 2

10.1136/bmjopen-2024-096098 online supplemental file 3

10.1136/bmjopen-2024-096098 online supplemental file 4
