# Supplementary material for: Randomised controlled trial with parallel process evaluation and health economic analysis to evaluate a nutritional management intervention, OptiCALS, for patients with amyotrophic lateral sclerosis: study protocol
Source: BMJ Open. 2025 May 27;15(5):e096098. doi: 10.1136/bmjopen-2024-096098 (PMC12121571; doi:10.1136/bmjopen-2024-096098)
Supplement: online supplemental file 1 [file bmjopen-15-5-s001.pdf]

The University Of Sheffield.

Trust local logo

NHS Sheffield Teaching Hospitals NHS Foundation Trust

OptiCALS

Participant Informed Consent Form

A randomised study of nutritional management in patients with Amyotrophic Lateral Sclerosis.

Participant Identification Number: ____________

		Initial each box
1.	I confirm that I have read and understand the information sheet dated [date] (Version [number]) for the above research study. I have had the opportunity to consider the information, ask questions and have had these answered satisfactorily.	☐
2.	I understand that my participation is voluntary and that I am free to withdraw at any time without giving any reason, without my medical care or legal rights being affected. If I do withdraw from the study for any reason, I understand that the team will not withdraw previously collected data, and that it may be used in the analysis.	☐
3.	I understand that relevant sections of my medical notes and data collected during the study may be looked at by individuals from the Sheffield Clinical Trials Research Unit, from regulatory authorities or from the NHS Trust, where it is relevant to my taking part in this research. I give permission for these individuals to have access to my records.	☐
4.	I understand that my responses will be kept strictly confidential. I give permission for members of the research team to have access to my responses. I understand that I will not be identified or identifiable in the report or reports that result from the research.	☐
5.	I agree that information collected by the research team, including a copy of this signed consent form, can be sent to and stored at the Sheffield Clinical Trials Research Unit for the purposes of monitoring and auditing.	☐
6.	I agree that data collected about me without personal identifiers may be used to support other research in the future, and may be shared with other researchers for comparison studies; and I give my permission for this.	☐
7.	I understand that my GP/MND Care Team will be informed of my participation in the trial. I agree to any necessary exchange of information between my GP and the research team. I understand that my GP/MND Care Team will be contacted should any concerns arise during the course of the trial.	☐
8.	I agree that my details will be shared with a postal / courier company for arranging equipment delivery / collection, and that they may be shared with an approved third party oral nutritional supplement (ONS) provider, for the purpose of allowing home delivery of ONS during the trial.	☐
9.	I understand how my data will be used in the study, including once my participation is over, if I decide to continue to use the OptiCALS website (if applicable).	☐
10.	I agree to take part in the above study.	☐
11.	I agree to my blood samples being stored and analysed beyond completion of the study. [OPTIONAL]	☐
12.	I consent to my caregiver (if applicable) being approached about taking part in the trial. I understand that my caregiver does not have to take part in the trial if they do not want to, and their decision will not affect my taking part. [OPTIONAL]	☐
13.	I understand that I may be invited to take part in two interviews about OptiCALS. I agree that researchers from the University of Sheffield who conduct these interviews can be provided with personal, identifiable, clinical information, collected by the clinical staff and the information stored in the OptiCALS website, to inform selection of participants and the subject matter of the interviews. If selected, I agree to participate in the interviews and for these to be recorded and transcribed verbatim. I understand that the recording will be destroyed at the end of the study; and, any quotations in reports about the research will be anonymous. [OPTIONAL]	☐
14.	I agree that my study visit may be recorded to ensure that the intervention is delivered as intended [OPTIONAL]	☐

Original for Trial Master File, 1 copy for participant and 1 copy for Site File.

15.	I agree that researchers can contact me regarding participation in other research in the future. I understand I will be provided with further information at that time and given the opportunity to decide whether or not to participate. [OPTIONAL]	☐
16.	I agree that routine data and survival status may still be collected should I decide to withdraw from the study. [OPTIONAL]	☐

N.B. Point 11-16 are optional. Please inform the member of the research team collecting your data if you do not want to consent to these options. You can still consent to be in the study if you choose not to consent to these points.

A randomised study to determine the benefits of nutritional management in patients with Amyotrophic Lateral Sclerosis.

i. If you would like to receive information about this research, and would like to be informed of the results at the end of the study, please tick the box corresponding to your preferred method of contact.

☐ **Post** ☐ **Email** ☐ **I do not want to be contacted**

To be completed by the participant / independent witness*:

Initial the box below:

I confirm that I have discussed the OptiCALS study with a member of the research team, prior to completing this form. ☐

Name of participant	Signature	d d	m m	y y y y

To be completed by the independent witness (if required):

Name of independent witness*	Signature	d d	m m	y y y y

To be completed by the researcher:

Initial each box:

I confirm that a discussion between the participant and a member of the research team has occurred prior to completing this form. ☐

Name of person taking consent	Signature	d d	m m	y y y y

If a independent witness is required (please initial):

I confirm that the independent witness is **NOT** the participating caregiver ☐

I confirm that the independent witness is **NOT** a member of the OptiCALS research team. ☐

Method of consent (please tick): ☐ **In person** ☐ **Verbal** ☐ **Postal** ☐ **Email / electronic**

*If participant is physically unable to sign and date to confirm consent, an independent witness can do this on their behalf. In such cases the witness should sign to confirm the participant has given consent to take part in the study.

Original for Trial Master File, 1 copy for participant and 1 copy for Site File.
