# Supplementary material for: Randomised controlled trial with parallel process evaluation and health economic analysis to evaluate a nutritional management intervention, OptiCALS, for patients with amyotrophic lateral sclerosis: study protocol
Source: BMJ Open. 2025 May 27;15(5):e096098. doi: 10.1136/bmjopen-2024-096098 (PMC12121571; doi:10.1136/bmjopen-2024-096098)
Supplement: online supplemental file 3 [file bmjopen-15-5-s003.pdf]

The University Of Sheffield.

Trust local logo

NHS Sheffield Teaching Hospitals NHS Foundation Trust

OptiCALS

Healthcare Professional Informed Consent Form - Interview

A randomised study of nutritional management in patients with Amyotrophic Lateral Sclerosis.

Participant Identification Number: ____________

		Initial each box
1.	I confirm that I have read and understand the information sheet dated [date] (Version [number]) for the above research study. I have had the opportunity to consider the information, ask questions and have had these answered satisfactorily.	☐
2.	I understand that my participation is voluntary and that I am free to withdraw at any time without giving any reason or my legal rights being affected. In addition, should I not wish to answer any particular question or questions, I am free to decline.	☐
3.	I understand that my responses will be kept strictly confidential. I give permission for members of the research team to have access to my responses. I understand that I will not be identified or identifiable in the report or reports that result from the research.	☐
4.	I agree that data collected about me without personal identifiers may be used to support other research in the future, and may be shared with other researchers for comparison studies; and I give my permission for this.	☐
5.	I agree to the interview being recorded and transcribed verbatim. I understand that the recording will be destroyed at the end of the study; and, any quotations in reports about the research will be anonymous.	☐
6.	I understand how my data will be used in the study.	☐
7.	I agree to take part in the interview.	☐
8.	I agree if invited, to being observed whilst delivering the intervention and being informally interviewed. [OPTIONAL]	☐
9.	I agree that study visits may be recorded to enable assessment of fidelity. [OPTIONAL]	☐

N.B. Points 8 and 9 are optional. Please inform the member of the research team collecting your data if you do not want to consent to these options. You can still consent to be in the study if you choose not to consent to these points.

If you would like to receive information about this research, and would like to be informed of the results at the end of the study, please tick the box corresponding to your preferred method of contact.

☐ **Post** ☐ **Email** ☐ **I do not want to be contacted**

To be completed by participant and researcher:

Participant: I confirm that I have discussed the OptiCALS study with a member of the research team, prior to completing this form. ☐

Person taking consent: I confirm that a discussion between the participant and a member of the research team has occurred prior to completing this form. ☐

Name of participant ____________ Signature ____________ d d / m m / y y y y

Name of person taking consent ____________ Signature ____________ d d / m m / y y y y

Original for Trial Master File, 1 copy for participant and 1 copy for Site File.
