# Supplementary material for: Randomised controlled trial with parallel process evaluation and health economic analysis to evaluate a nutritional management intervention, OptiCALS, for patients with amyotrophic lateral sclerosis: study protocol
Source: BMJ Open. 2025 May 27;15(5):e096098. doi: 10.1136/bmjopen-2024-096098 (PMC12121571; doi:10.1136/bmjopen-2024-096098)
Supplement: online supplemental file 4 [file bmjopen-15-5-s004.pdf]

The University Of Sheffield.

Trust local logo

NHS Sheffield Teaching Hospitals NHS Foundation Trust

OptiCALS

Informed Consent Form - Participant Observations

A randomised study of nutritional management in patients with Amyotrophic Lateral Sclerosis.

Participant Identification Number: ____________________

Initial each box

1.	I confirm that I have read and understand the information sheet dated [date] (Version [number]) for the above research study. I have had the opportunity to consider the information, ask questions and have had these answered satisfactorily.	☐
2.	I understand that my participation is voluntary and that I am free to withdraw at any time without giving any reason or my legal rights being affected. If I do withdraw from the study for any reason, I understand that the team will not withdraw previously collected data, and that it may be used in the analysis.	☐
3.	I understand that my responses will be kept strictly confidential. I give permission for members of the research team to have access to my responses. I understand that I will not be identified or identifiable in the report or reports that result from the research.	☐
4.	I agree to my study visit being observed by a member of the research team in order to ensure that the intervention is delivered as intended.	☐
5.	I agree to my study visit being recorded and transcribed verbatim. I understand that the recordings will be destroyed at the end of the study; and, any quotations in reports about the research will be anonymous.	☐
6.	I agree that information collected by the research team, including a copy of this signed consent form, can be sent to and stored at the Sheffield Clinical Trials Research Unit for the purposes of monitoring and auditing.	☐
7.	I agree that data collected about me without personal identifiers may be used to support other research in the future, and may be shared with other researchers for comparison studies; and I give my permission for this.	☐
8.	I understand how my data will be used in the study	☐

To be completed by participant / witness* and researcher:

Participant or witness*: I confirm that I have discussed the OptiCALS study with a member of the research team, prior to completing this form. ☐

Person taking consent: I confirm that a discussion between the participant and a member of the research team has occurred prior to completing this form. ☐

Name of participant or witness*	Signature	d d m m y y y y
Name of person taking consent	Signature	d d m m y y y y

Original for Trial Master File, 1 copy for participant and 1 copy for Site File.

*If participant is physically unable to sign and date to confirm consent, a witness can do this on their behalf. If a witness is not present, ask the participant to check the box to confirm the study has been explained to them.

**If the participant is unable to sign or check the box please sign to confirm that verbal consent has been given and that the patient has agreed to be in the study.
