# Supplementary material for: Randomised controlled trial with parallel process evaluation and health economic analysis to evaluate a nutritional management intervention, OptiCALS, for patients with amyotrophic lateral sclerosis: study protocol
Source: BMJ Open. 2025 May 27;15(5):e096098. doi: 10.1136/bmjopen-2024-096098 (PMC12121571; doi:10.1136/bmjopen-2024-096098)
Supplement: Supplementary file 5 [file bmjopen-2024-096098.reviewer_comments.pdf]

PEER REVIEW HISTORY

BMJ Open publishes all reviews undertaken for accepted manuscripts. Reviewers are asked to complete a checklist review form and are provided with free text boxes to elaborate on their assessment. These free text comments are reproduced below.

ARTICLE DETAILS

Title (Provisional)

A Randomised Controlled Trial with parallel process evaluation and health economic analysis to evaluate a nutritional management intervention, OptiCALS, for patients with Amyotrophic Lateral Sclerosis: Study Protocol

Authors

Peace, Arron Peace; White, David Alexander; Hackney, Gemma; Bradburn, Mike; Norman, Paul; White, Sean; Al-Chalabi, Ammar; Baird, Wendy; Beever, Daniel; Cade, Janet; Coates, Elizabeth; Cooper, Cindy; Ezaydi, Naseeb; Halliday, Vanessa; Shaw, Pamela J; Stavroulakis, Haris; Waterhouse, Simon; Young, Tracey Anne; McDermott, Christopher J; Maguire, Chin; Group, HighCALS

This article was not externally reviewed at BMJ Open. Protocols that have been independently assessed prior to submission to BMJ Open are usually fast-tracked to publication on the grounds that further substantial changes will not be possible. This independent assessment will usually be external, independent review for both a substantial grant award from a non-commercial or government funder and ethics approval.
